# The effect of single versus multiple piezocisions on the rate of canine retraction: a randomized controlled trial

**DOI:** 10.1186/s12903-024-04716-6

**Published:** 2024-08-30

**Authors:** Farah Y. Eid, Ahmed R. El-Kalza

**Affiliations:** Department of Orthodontics, Faculty of Dentistry, Alexandria University, Champolion street, Azarita, Alexandria, Egypt

**Keywords:** Piezocision, Canine retraction, Acceleration, Anchorage loss, Root resorption, Tipping movement

## Abstract

**Background:**

Piezocision is a minimally invasive surgical method aiming to accelerate tooth movement. However, its effect was found to be transient, appertaining to the regional acceleratory phenomenon (RAP). Hence, the aim of the study was to evaluate the effect of single and multiple piezocisions on the rate of orthodontic tooth movement (OTM). Moreover, the impact of both protocols on canine tipping and orthodontically induced inflammatory root resorption (OIIRR) has been assessed.

**Methods:**

Thirty indicated patients for the therapeutic extraction of maxillary first premolars were enlisted in this split-mouth study, and they were randomly split into two equal groups, each including 15 subjects. In the Single Application Group (SAG), one side of the maxillary arch arbitrarily received a single piezocision before the onset of canine retraction, whereas in the Multiple Application Group (MAG), piezocisions were randomly performed on one side, three times on a monthly basis, over the 12-week study period. The contralateral sides of both groups served as the controls. Canine retraction was carried out bilaterally using nickel-titanium closed-coil springs, delivering 150 g of force, and the rate of tooth movement, as well as canine tipping were evaluated on a monthly basis, over a 3-month period. Cone-bean computed tomography scans were also conducted pre- and post- canine retraction, and OIIRR was assessed using Malmgren Index.

**Results:**

The reported outcomes revealed a significant increase in the amount of canine retraction, canine tipping, as well as root resorption scores on the experimental sides in both groups SAG and MAG post-retraction (*p* < 0.001). However, upon comparing the experimental sides in both groups, non-significant differences have been observed between them regarding all the assessed outcomes (*p* > 0.05).

**Conclusions:**

Single and multiple piezocisions effectively accelerate OTM in comparison to conventional orthodontic treatment, with relative outcomes reported by both intervention frequencies. Accordingly, single piezocision is recommended as an adjunct to OTM. Furthermore, significant tooth tipping as well as a significantly higher root resorption risk accompanies both single and multiple piezocision applications in conjunction with OTM.

**Name of the Registry:**

Clinicaltrials.gov

**Trial Registration Number:**

NCT05782088

**Date of Registration:**

23/03/2023 “Retrospectively registered”.

**URL:**

https://clinicaltrials.gov/ct2/show/NCT05782088

## Background

The prolonged orthodontic treatment period is usually a matter of serious concern for the patients, and it also results in several dental and periodontal side effects [1–3]. Moreover, the prolonged treatment duration has an adverse effect on patient compliance, and his/her willingness to continue treatment. Consequently, several methods aiming to accelerate orthodontic tooth movement (OTM) and lessen the treatment duration have been proposed, including surgical [4, 5] and non-surgical adjuncts [6].

The suggested acceleratory surgical interventions include corticotomy, which is considered a significantly invasive technique, since it involves the elevation of a relatively large flap, followed by cortical bone cuts, all of which might result in post-operative complications [4, 7, 8]. Several other less invasive surgical methods have been proposed for the acceleration of OTM, such as micro-osteoperforations [9, 10], corticison [5, 11], as well as piezocision [12, 13].

Piezocision is known to involve minor piezoelectric cuts without flap elevation [14, 15]. The acceleratory effect of piezocision and all the other surgical methods is mainly credited to the regional acceleratory phenomenon (RAP), which involves a transient demineralization, together with a surge in cellular activity in the alveolar bone, in response to the cortical bone injury [16]. The impact of RAP has been reported to be temporary, and entirely reversible. Moreover, the magnitude of RAP was found to be dependent on the corticotomy depth [17].

The impact of piezocision on the rate of OTM has been investigated in several trials, and despite its effectiveness in accelerating tooth movement, its effect was found to be transient [18, 19], which might be related to the temporary nature of RAP as previously explained. Therefore, it has been suggested that performing multiple piezocisions throughout the treatment might be helpful in prolonging and/or re-inducing the biological effect of RAP, and accordingly, maintain the acceleratory effect for a longer time [20, 21].

With orthodontically induced inflammatory root resorption (OIIRR) being a commonly encountered iatrogenic repercussion of orthodontic treatment, the impact of the proposed methods for acceleration of OTM has been assessed regarding this issue, based on the rationale of decreasing treatment time could concurrently decrease the incidence or the severity of OIIRR. Accordingly, piezocision and OIIRR with OTM have been tested in several studies with contradictory findings being reported [22–24].

In conclusion, by reviewing the literature, no clinical trial has been conducted to investigate the impact of multiple piezocisions on the rate of tooth movement, except for one study employing an initial corticotomy followed by a second flapless corticotomy using piezosurgery after two months, to facilitate the traction of an impacted canine [25]. Therefore, the aim of our study was to assess and compare the effect of single versus multiple piezocisions on the rate of OTM, judged by the rate of canine retraction. Moreover, maxillary canine tipping, and OIIRR with both single and multiple piezocision techniques were evaluated pre- and post- canine retraction.

The null hypothesis was that there are no significant differences in the rate of canine retraction, the amount of experienced canine tipping during movement, and in the risk of OIIRR with both single and multiple piezocisions.

## Materials and methods

### Study design

The study was a compound design randomized controlled trial, comprising two parallel groups, with a split-mouth design in each.

### Study subjects

Thirty participants with an age ranging from 15 to 25 years, have been appointed for this study. The sample size was calculated based on 95% confidence level to detect differences in the canine retraction rate with and without piezocision. Alfawal et al [15] reported that the mean ± SD canine retraction rate at the third month = 1.10 ± 0.29 mm/month on the piezocision side, and 0.98 ± 0.22 mm/month on the control side. The mean ± SD difference = 0.11 ± 0.255, and 95% confidence interval= -0.04, 0.26. Repeated piezocision is assumed to accelerate orthodontic tooth movements [20]. Based on comparison of paired means, the minimum sample size was calculated to be 14 per group, increased to 15 to make up for cases lost to follow-up. The total required sample size = number of groups × number per group = 2 × 15 = 30 patients [26]. The sample was calculated using MedCalc Statistical Software version 19.0.5 (MedCalc Software bvba, Ostend, Belgium; https://www.medcalc.org; 2019).

Ethical approval has been procured from the Institutional Review Board of the Faculty of Dentistry, Alexandria University, Alexandria, Egypt (IRB:00010556–IORG:0008839). Manuscript Ethics Committee number 0582-01/2023. Patients were recruited from the outpatient clinic, Department of Orthodontics, Alexandria University starting January 2023, and the study was terminated in January 2024. Subjects were examined and screened, and the following enrolment criteria have been considered: (1) Class I bimaxillary protrusion, and Class II division 1 patients requiring the extraction of maxillary 1st premolars with consequent canine retraction, (2) Healthy systemic condition with no chronic problems, (3) No previous orthodontic treatment, (4) Acceptable oral hygiene, (5) Healthy periodontium. The study procedures were thoroughly explained to all the enrolled subjects, and signed informed consents were attained accordingly. All research procedures were performed in agreement with the relevant guidelines and regulations, as stated in the Declaration of Helsinki.

### PICO

Patients requiring the therapeutic extraction of maxillary 1st premolars bilaterally (P), were tested for piezocision along with canine retraction (I), applied only once versus multiple times (C), to evaluate the difference in the rate of tooth movement with both protocols (O).

### Randomization and subject allocation

The thirty recruited patients were randomly allocated to either group (15 per group), using a computer-generated randomization code (Sealed Envelope Ltd). As per the split-mouth design within each group, the randomization process was repeated once again for allocation of the “experimental” and “control” sides in the maxillary arch. Randomization was performed by a trial independent person.

### Procedure

#### Patients’ preparation

Preparation for fixed orthodontic treatment entailed recording the enrolled subjects’ medical and dental history, along with collecting the customary orthodontic records (photographs, x-rays, and study models). Oral hygiene reinforcement was also mandatory prior to the onset of orthodontic treatment. Maxillary and mandibular straight wire fixed Roth appliances (Sprint^®^ II; Forestadent, Germany) were bonded by the same operator, with 0.022$\:\:\times\:\:$0.028-inch slots in all participants, after which they were referred for maxillary first premolars’ extraction. Levelling and alignment were then started, and a wire sequence of 0.014-inch, 0.018-inch, followed by 0.016$\:\:\times\:\:$0.022-inch NiTi wires were used, over an approximate period of 3–4 months. This stage was considered achieved when a 0.016$\:\:\times\:\:$0.022-inch stainless steel arch wire was positioned passively in all the maxillary teeth, on which canine retraction will be performed.

#### Anchorage preparation

After levelling and alignment, anchorage reinforcement was ensured through the bilateral inter-radicular placement of mini-screws between the maxillary second premolars and first molars, 8 mm from the apex of the interdental papilla. The placed mini-screws were 1.7 mm in diameter, and 8 mm in length (Orthoeasy; Forestadent, Germany). Mini-screws were installed under local anesthesia, with a screw driver employed for the self-drilling process.

#### Intervention

In group SAG, piezocision was performed only once prior to the commencement of canine retraction (T0) on one side of the maxillary arch that has been randomly selected. As for the MAG group, piezocision was repeated three times, on a monthly basis (T0, T1, and T2), over the 12-week study period. The contralateral sides in both groups represented the controls.

**Fig. 1:**
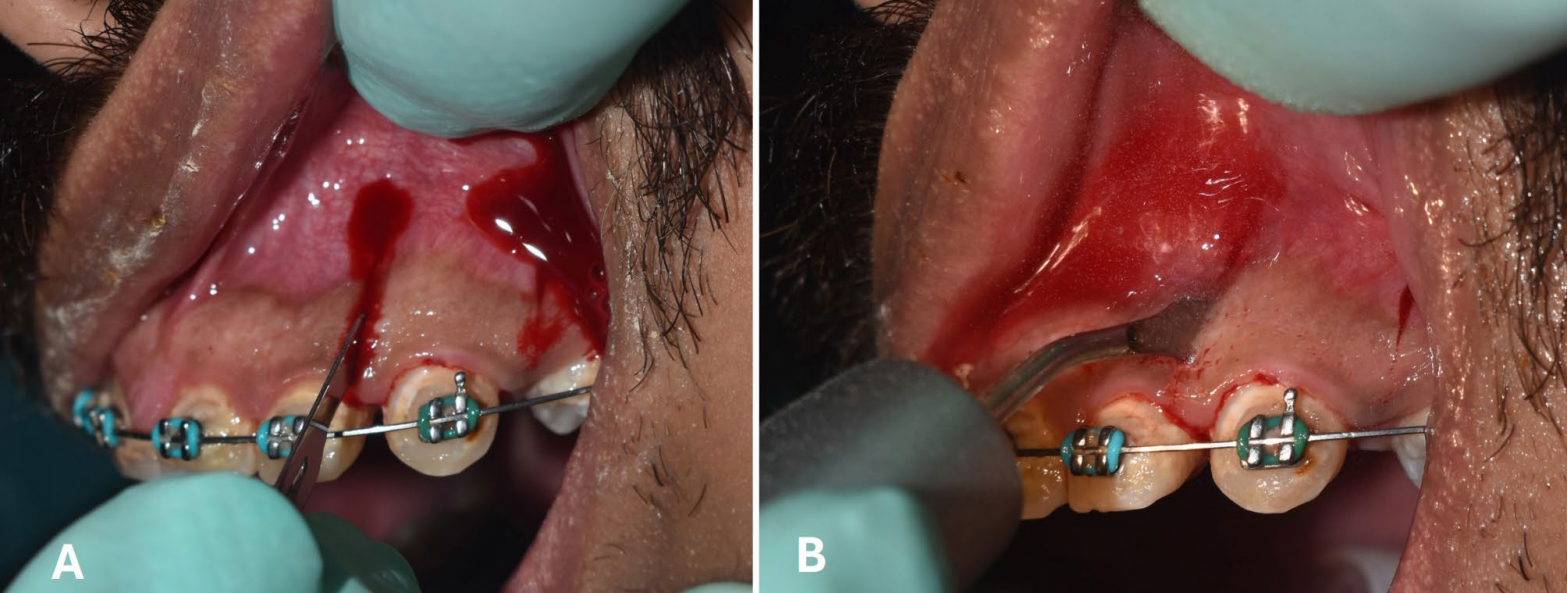
**(A)** Vertical interproximal incisions mesial and distal to the maxillary canine using surgical blade No. 15. **(B)** Vertical cortical cuts using the Piezo surgical knife, with a depth of 3 mm

On the experimental sides in both groups, the surgical procedure was conducted under local infiltrative anaesthesia to the mesial and distal sides of the maxillary canine. Vertical interproximal incisions were made 5 mm apical to the mesial and distal interdental papilla of the experimental canine, on the buccal aspect using surgical blade No. 15. Incisions extended apically 10 mm in length through the periosteum, permitting the blade to reach the alveolar bone. A Piezo surgical knife (Piezomed, tip B1) was subsequently employed to create the cortical bone incision through the gingival opening, to an approximate depth of 3 mm **(**Fig. 1**)**. No suturing has been required for the soft tissue incisions after termination of the surgery [18, 27]. Moreover, analgesics (paracetamol) were prescribed post-operatively, whereas anti-inflammatory drugs were prohibited to avoid intervening with the RAP [18].

**Fig. 2:**
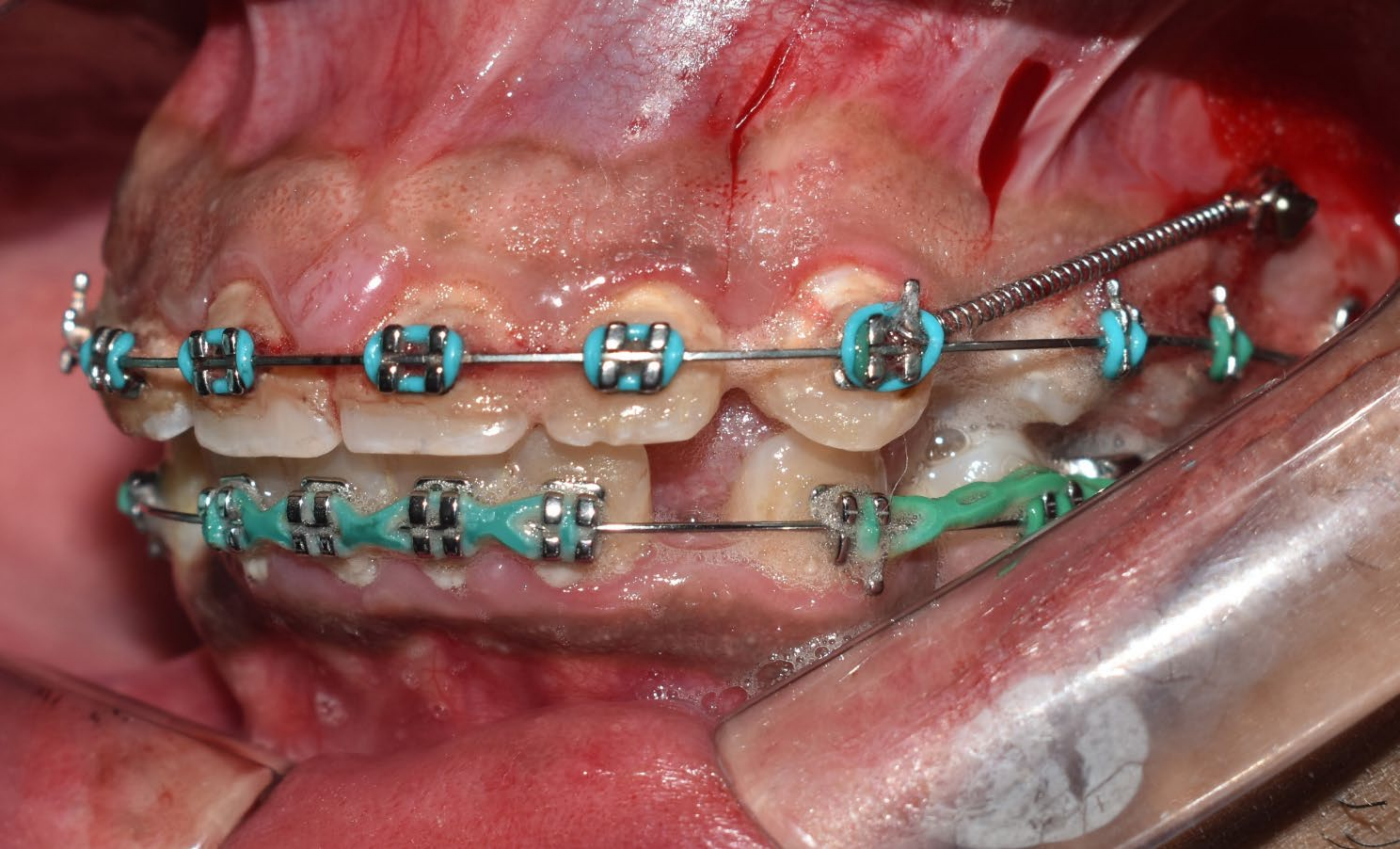
Canine retraction using NiTi closed-coil springs bilaterally stretched between the mini-screw head and the canine bracket hook

Canine retraction was accomplished using nickel-titanium (NiTi) closed-coil springs stretched between the canine bracket hook and the mini-screw head, with a force of 150 g applied on each side of the maxillary arch, and the force magnitude was adjusted each visit, as measured by a force gauge (Morelli Ortodontia, Brazil) (Fig. 2).

### Blinding

Due to the nature of the clinical procedure, neither the patient nor the operator could be blinded during the intervention. However, both the operators during measurements, and the statistician during data analysis were blinded.

### Outcomes

**Fig. 3:**
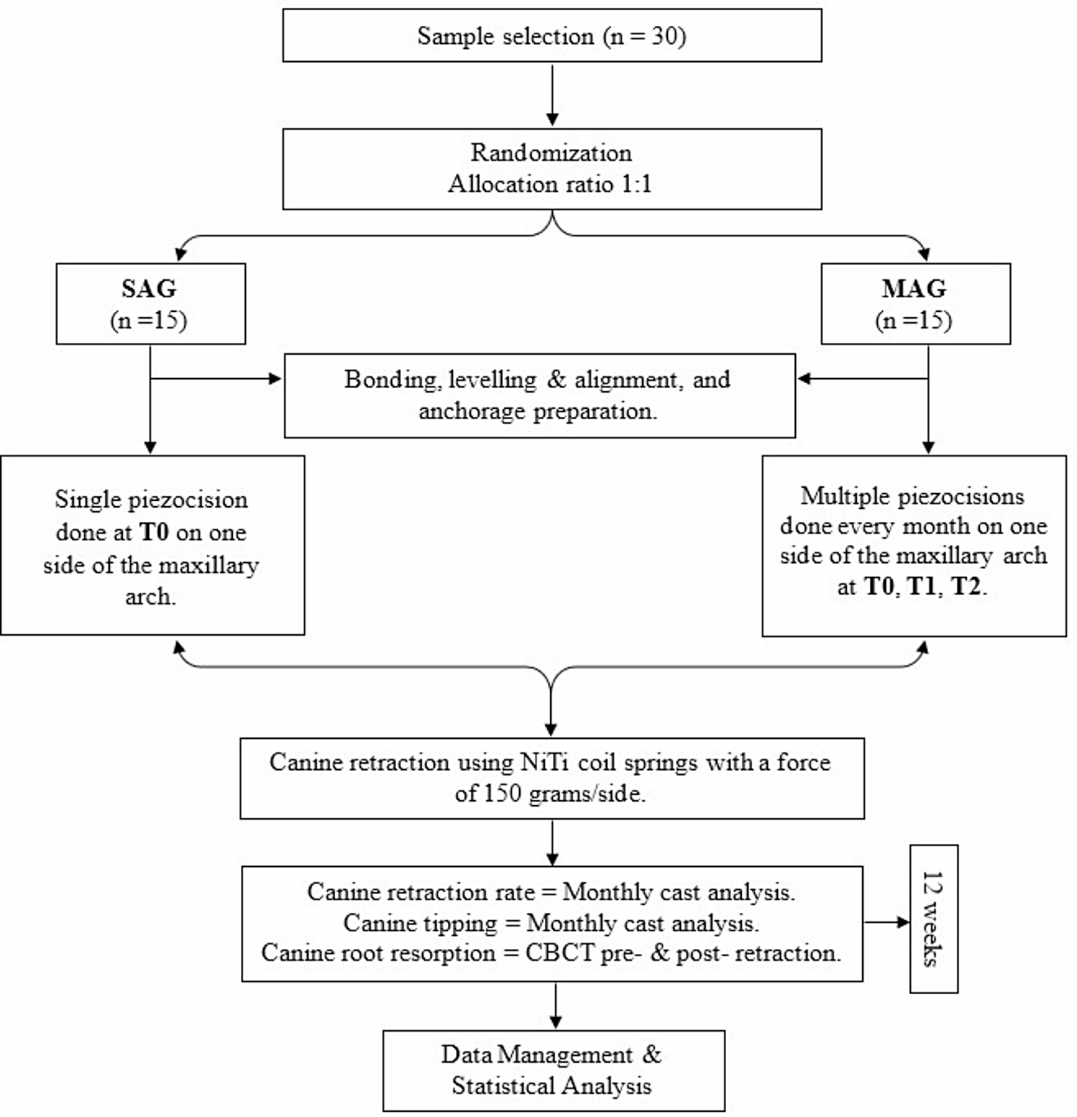
Research design flowchart summarizing the study procedures

Alginate impressions (Ca37; Cavex, Haarlem, The Netherlands) were taken before the start of canine distalization (T0), and then repeated on a monthly basis (T1, T2, T3) over the 12-week research duration. Dental models were then poured, coded, and scanned (inEos X5 CAD/CAM lab scanner; Dentsply Sirona, PA, USA), producing three-dimensional (3D) digital images of the fabricated models. The needed measurements were carried out using AutoCAD version 2020 (AutoCAD; Autodesk, USA). Pre-retraction and post-retraction cone-beam computed tomography (CBCT) scans were also conducted in both groups SAG and MAG (within a 12-week interval). A research design flowchart is represented in Fig. 3, recapitulating the research methodology.

### Measurement of canine retraction

**Fig. 4:**
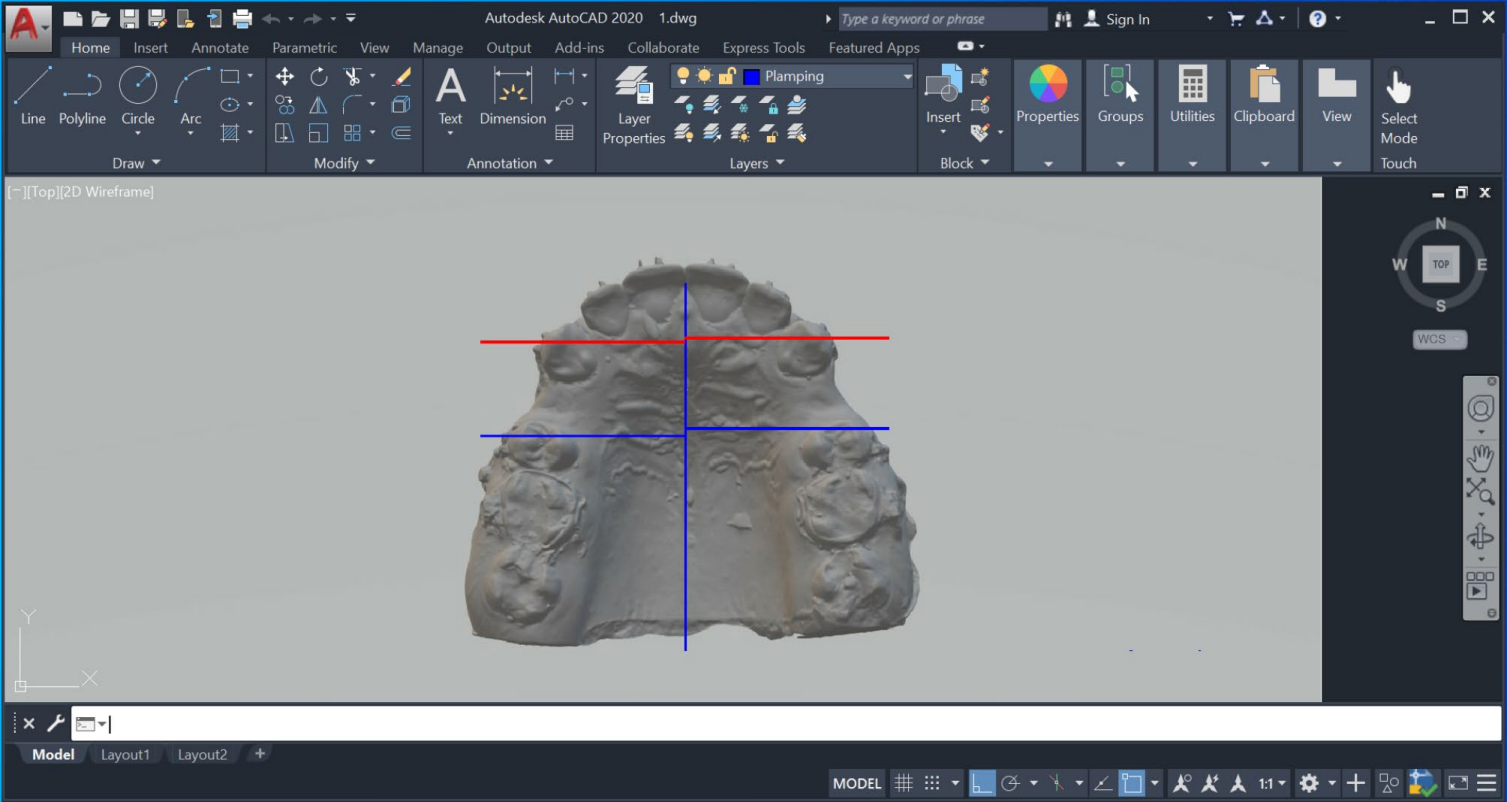
Measurement of canine retraction using AutoCAD between the canine cusp tips and the medial ends of the third rugae

Various landmarks were determined on the maxillary arch, including the mid-palatal raphe, the medial points on the third right and left rugae, and the cusp tips of the right and left maxillary canines. From both the medial points of the right and left third rugae, and the cusp tips of the right and left maxillary canines, perpendicular lines were dropped to the mid-palatal raphe. Antero-posterior measurements were subsequently performed between the canine lines and the third rugae lines on each side, for the assessment of the canine retraction rate [28] (Fig. 4).

### Measurement of canine tipping

**Fig. 5:**
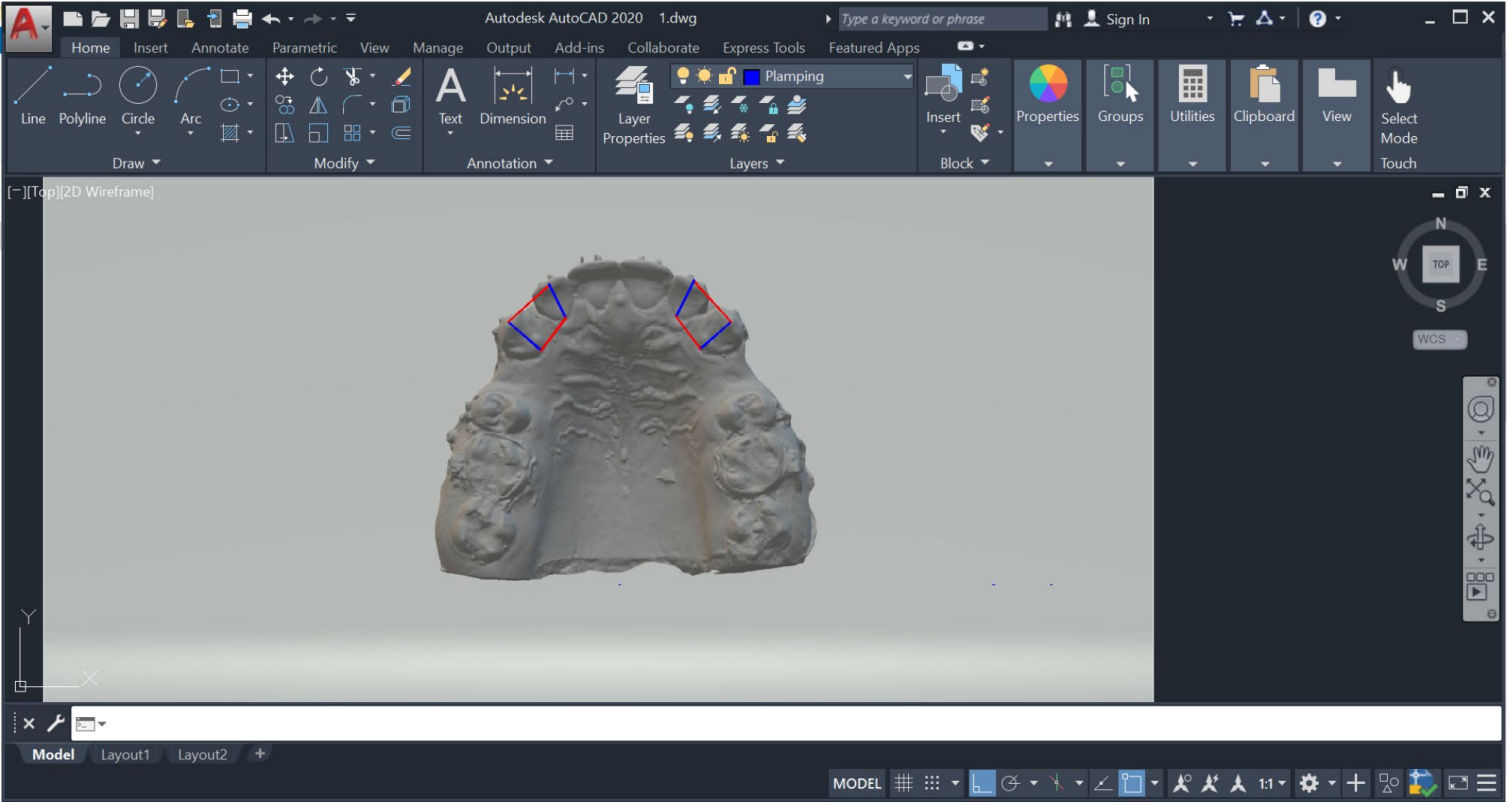
Evaluation of canine tipping using AutoCAD by measuring the distance between the canine and lateral incisor at the incisal and cervical levels

Tipping of the maxillary canine during retraction was evaluated by drawing vertical lines on the palatal surfaces of the lateral incisor and the canine that extend from the middle of the incisal edge of the lateral incisor and the cusp tip of the maxillary canine to the middle of the cervical line of each, thereby dividing each of them into equal halves. The distance between the lateral incisor and the canine was assessed at two points on their clinical crowns: incisal, and cervical, enabling the detection of crown tipping of the canine during distalization, if there was a difference in the measurements between both the assessed levels [9] (Fig. 5).

### Measurement of OIIRR

In both groups, maxillary canine root resorption was evaluated on the procured pre-retraction and post-retraction CBCT scans, that were conducted using the J. Morita R100 Cone beam 3D Imaging System machine (MFG Corp., Kyoto, Japan). The scan was executed with a Field of View (FOV) of 100 × 50 mm (Width × Height). Volumes’ reconstruction was carried out with a 0.160 mm isometric voxel size, a tube voltage of 90 kVp and 8 mA, and an exposure time of 20 s.

**Fig. 6:**
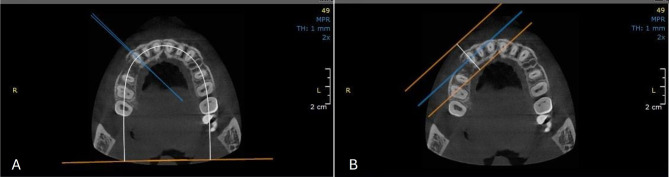
Axial views showing the adjusted focal trough permitting the sectioning of the right maxillary canine with an interval of 0.1 mm in two directions: **(A)** Labiolingual, **(B)** Mesiodistal

**Fig. 7:**
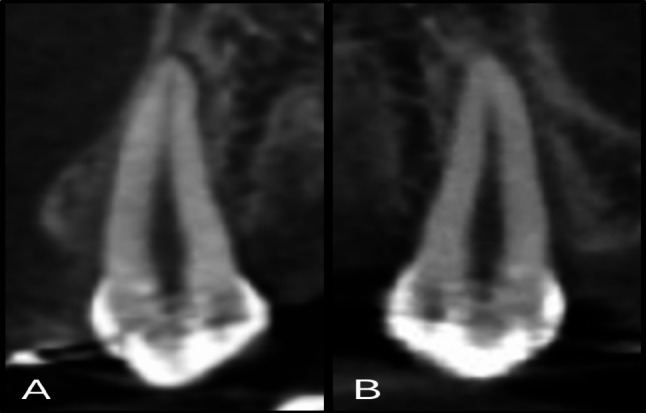
The re-oriented CBCT image of the maxillary canine revealing the maximum root length. **(A)** Labiolingual cross-section, **(B)** Mesiodistal cross-section

Malmgren Index [29] was used for the assessment of OIIRR, and each of the tested canines was given a score ranging from 0 to 4, according to the degree of detected resorption. Using the software OnDemand3D**™** (Cybermed Inc., South Korea), and utilizing the arch section module, the focal trough was adjusted twice to allow the mesiodistal and the labiolingual sectioning of each canine, parallel to the long axis of its root (Fig. 6). The chosen slice thickness interval was 0.1 mm. The two perpendicular cross-sections showing the maximum length of the canine root were subsequently selected for evaluation using the designated index [30] (Fig. 7).

### Intra-rater and inter-rater reliability

Calibration on the study measurements was performed for two assessors (F.E., and A.E.), who repeated the measurements to ensure consistency, within a one-week interval. Both intra- and inter-rater reliability were evaluated, and intraclass correlation coefficient (ICC) ranged from 0.87 to 0.99 indicating excellent reliability between examiners and across time [31].

### Statistical analysis

Normality was tested for the included variables using descriptive statistics, plots (Q-Q plots and histograms), and Shapiro Wilk normality tests. All quantitative data exhibited normal distribution, so means and standard deviation (SD) were calculated, and parametric tests were implemented. Comparisons of canine retraction and tipping between the two groups (single vs. multiple piezocision) were performed using independent samples t-test, while comparisons between the experimental and control sides were performed using paired samples t-test. Mean differences and 95% confidence intervals were calculated. Meanwhile, comparisons between different time points were performed through repeated measures ANOVA, followed by multiple pairwise comparisons with Bonferroni adjusted significance levels. Comparisons of root resorption scores between the two groups (single vs. multiple piezocision) were done using Mann-Whitney U test, whereas comparisons between the experimental and control sides, and between the pre- and post- scores were performed using Wilcoxon Signed Ranks test. The significance level was set at p-value < 0.05. Data analysis was performed using IBM SPSS for Windows (Version 26.0).

## Results

Over the study period, no subject dropouts were recorded neither in the pre-intervention period, nor throughout the remainder of the research duration. Non-significant differences have been reported between the enrolled participants in the two study groups regarding their baseline characteristics (*p* > 0.05), as displayed in Table 1. No mini-screw failures have also been reported in any of the study participants over the 12-week observation period. Moreover, all the study models that were obtained every month, as well as the pre- and post-retraction CBCT scans were accounted for.

**Table 1:** Demographic characteristics of the two study groups

		SAG	MAG	*P* value
Age: mean (SD) a		17.33 (1.88)	17.40 (1.92)	0.92
Gender: n (%) b	Male	9 (60%)	7 (46.7%)	0.46
	Female	6 (40%)	8 (53.3%)	

*a* Independent samples t-test, *b* Chi-square test

### Canine retraction

The amount of maxillary canine distalization at the studied time points is depicted in Table 2, regarding both groups SAG and MAG. In group SAG, the mean distance travelled by the canines has been significantly greater on the experimental sides in comparison with the control sides at all the assessed time points (*p* < 0.001), in addition to the total moved distance after the 12-week study period, with that being 4.49 mm ± 0.34 on the experimental side, and 2.77 mm ± 0.29 on the control side. Moreover, on the experimental side, the amount of the canine retraction was significantly less at T3 when compared to those recorded at T1 and T2 (*p* < 0.01). Opposingly, on the control side, non-significant differences have been observed in the amount of retraction across time (*p* = 0.09).

**Table 2:** Comparison of canine retraction (mm) between the experimental and the control sides in groups SAG and MAG

		Experimental	Control	Mean difference (95% CI)	*P* value #
		Mean (SD)			
SAG	T1	1.65 (0.17) A	0.93 (0.13)	0.72 (0.67, 0.76)	< 0.001*
	T2	1.62 (0.16) A	0.92 (0.10)	0.70 (0.62, 0.78)	< 0.001*
	T3	1.23 (0.09) B	0.92 (0.12)	0.31 (0.22, 0.40)	< 0.001*
	Total	4.49 (0.34)	2.77 (0.29)	1.73 (1.58, 1.87)	< 0.001*
	P value †	< 0.001*	0.90		
MAG	T1	1.74 (0.15) A	0.92 (0.12)	0.83 (0.78, 0.88)	< 0.001*
	T2	1.72 (0.15) A	0.92 (0.09)	0.80 (0.74, 0.86)	< 0.001*
	T3	1.22 (0.10) B	0.95 (0.10)	0.26 (0.18, 0.35)	< 0.001*
	Total	4.68 (0.27)	2.79 (0.29)	1.89 (1.78, 2.01)	< 0.001*
	P value †	< 0.001*	0.12		

*SD* Standard Deviation, *CI* Confidence Interval

#: Paired samples t-test was used

†: Repeated measures ANOVA was used

A, B: Different letters denote statistically significant differences between time points within experimental sides

In group MAG, a similar pattern to that observed in the SAG has been documented, with the experimental sides showing statistically greater moved distances by the maxillary canines at T1, T2, and T3 in comparison with the control sides (*p* < 0.001). Also, the total amount of canine retraction achieved on the experimental side was statistically higher than that recorded on the control side, with values of 4.68 mm ± 0.27, and 2.79 mm ± 0.29, respectively. The experimental side showed a significant reduction in the moved distances by the canines at T3 in comparison with both T1 and T2 (*p* < 0.001), whereas the control side revealed a relatively constant rate of tooth movement across the three time points (*p* = 0.12).

**Fig. 8:**
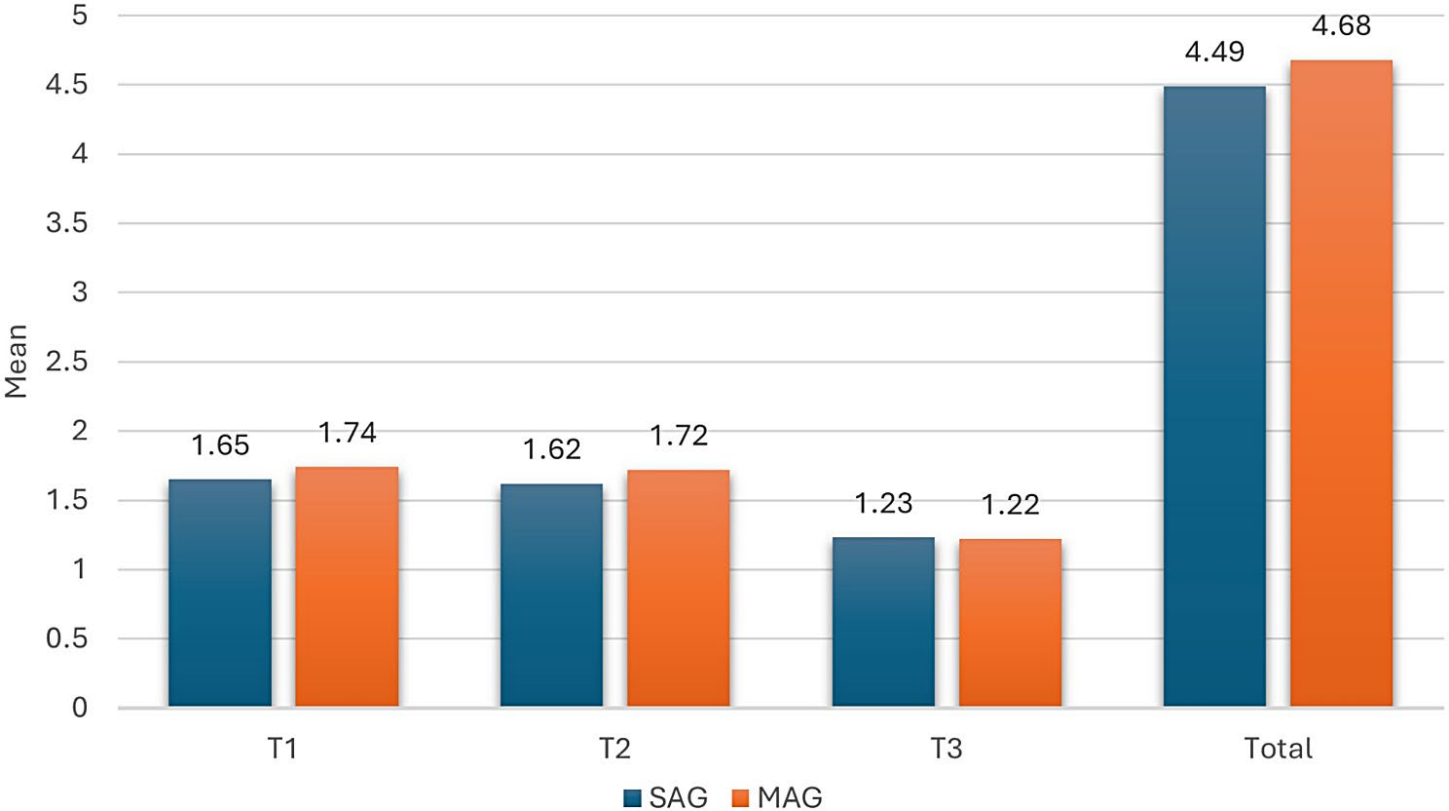
Comparison of canine retraction on the experimental sides in groups SAG and MAG

When the experimental sides in both groups SAG and MAG were compared as presented in Fig. 8, non-significant differences between both groups have been documented at T1 (*p* = 0.11), T2 (*p* = 0.10), T3 (*p* = 0.68), as well as in the total moved distance after 12 weeks (*p* = 0.11). However, a significantly less amount of canine retraction was recorded at T3 in comparison to T1, and T2 (*p* < 0.001).

### Canine tipping

Canine tipping on both the experimental and control sides in groups SAG and MAG is displayed in Table 3. In the two study groups, a similar trend has been observed on both the experimental and control sides, where a significant increase in the moved distance by the maxillary canine has been documented relative to the lateral incisor at the incisal, and cervical levels, as well as after calculating the difference between both levels at all the assessed time points (*p* < 0.001). Furthermore, in both single and multiple application groups, a statistically greater amount of maxillary canine tipping has been noted on the experimental sides relative to the control sides at all the measured levels (incisal, cervical, difference between both), and at all the evaluated time points aside from the baseline (T0) (*p* < 0.05).

**Table 3:** Comparison of canine tipping between the experimental and the control sides in groups SAG and MAG

			Experimental	Control	Mean difference (95% CI)	*P* value 1
			Mean (SD)			
SAG	Incisal	T0	0.95 (0.09) A	0.96 (0.12) A	-0.02 (-0.05, 0.02)	0.31
		T1	2.46 (0.22) B	1.95 (0.29) B	0.51 (0.34, 0.68)	< 0.001*
		T2	3.95 (0.34) C	3.19 (0.75) C	0.76 (0.36, 1.16)	0.001*
		T3	5.54 (0.49) D	4.60 (1.15) D	0.94 (0.43, 1.45)	0.001*
		T3-T0	4.59 (0.46)	3.63 (1.09)	0.96 (0.45, 1.47)	0.001*
		P value 2	< 0.001*	< 0.001*		
	Cervical	T0	0.78 (0.07) A	0.77 (0.09) A	0.01 (-0.04, 0.06)	0.66
		T1	2.16 (0.19) B	1.73 (0.28) B	0.43 (0.24, 0.61)	< 0.001*
		T2	3.49 (0.30) C	2.81 (0.70) C	0.69 (0.29, 1.09)	0.003*
		T3	4.94 (0.48) D	4.11 (1.11) D	0.83 (0.32, 1.34)	0.003*
		T3-T0	4.16 (0.45)	3.34 (1.11)	0.82 (0.32, 1.32)	0.004*
		P value 2	< 0.001*	< 0.001*		
	Difference	T0	0.16 (0.10) A	0.19 (0.08) A	-0.03 (-0.07, 0.01)	0.15
		T1	0.31 (0.07) B	0.22 (0.10) B	0.08 (0.02, 0.15)	0.01*
		T2	0.45 (0.09) C	0.38 (0.09) C	0.07 (0.05, 0.10)	< 0.001*
		T3	0.60 (0.05) D	0.48 (0.06) D	0.11 (0.09, 0.13)	< 0.001*
		T3-T0	0.43 (0.08)	0.29 (0.05)	0.14 (0.09, 0.19)	< 0.001*
		P value 2	< 0.001*	< 0.001*		
MAG	Incisal	T0	0.94 (0.11) A	0.96 (0.10) A	-0.03 (-0.08, 0.02)	0.23
		T1	2.29 (0.25) B	1.82 (0.27) B	0.47 (0.33, 0.60)	< 0.001*
		T2	3.78 (0.53) C	3.42 (0.55) C	0.36 (0.18, 0.90)	0.02*
		T3	5.35 (0.43) D	4.68 (0.67) D	0.67 (0.30, 1.04)	0.002*
		T3-T0	4.41 (0.51)	3.71 (0.73)	0.70 (0.34, 1.05)	0.001*
		P value 2	< 0.001*	< 0.001*		
	Cervical	T0	0.73 (0.09) A	0.78 (0.09) A	-0.05 (-0.10, 0.002)	0.06
		T1	2.04 (0.21) B	1.45 (0.21) B	0.59 (0.46, 0.71)	< 0.001*
		T2	3.26 (0.42) C	3.00 (0.54) C	0.25 (0.20, 0.71)	0.02*
		T3	4.69 (0.50) D	4.19 (0.64) D	0.50 (0.08, 0.92)	0.02*
		T3-T0	3.96 (0.54)	3.41 (0.69)	0.55 (0.13, 0.98)	0.02*
		P value 2	< 0.001*	< 0.001*		
	Difference	T0	0.21 (0.05) A	0.18 (0.08) A	0.02 (-0.02, 0.06)	0.29
		T1	0.25 (0.13) B	0.37 (0.19) B	-0.12 (-0.23, -0.01)	0.03*
		T2	0.52 (0.29) C	0.42 (0.12) C	0.10 (0.08, 0.29)	0.02*
		T3	0.66 (0.31) D	0.49 (0.12) D	0.17 (0.02, 0.36)	0.01*
		T3-T0	0.45 (0.33)	0.31 (0.08)	0.15 (0.04, 0.33)	0.01*
		P value 2	< 0.001*	< 0.001*		

*SD* Standard Deviation, *CI* Confidence Interval

P value 1: Paired samples t-test was used

P value 2: Repeated measures ANOVA was used

*Statistically significant at p value < 0.05

A-D: Different letters denote statistically significant differences between time points within each group

In Table 4, comparisons between the amount of resultant canine tipping post-retraction on the experimental sides in groups SAG and MAG are displayed. Statistically non-significant differences have been reported between both groups at all the assessed levels and time points (*p* > 0.05). Within group comparisons showed statistically significant differences in the amount of canine tipping at all the time points, and at all the evaluated levels (*p* < 0.001).

**Table 4:** Comparison of canine tipping between the experimental sides in both groups SAG and MAG

		SAG	MAG	Mean difference (95% CI)	*P* value #
		Mean (SD)			
Incisal	T0	0.95 (0.09) A	0.94 (0.11) A	0.01 (-0.06, 0.08)	0.75
	T1	2.46 (0.22) B	2.29 (0.25) B	0.18 (-0.002, 0.35)	0.06
	T2	3.95 (0.34) C	3.78 (0.53) C	0.17 (-0.17, 0.50)	0.32
	T3	5.54 (0.49) D	5.35 (0.43) D	0.19 (-0.15, 0.54)	0.26
	Difference (T3-T0)	4.59 (0.46)	4.41 (0.51)	0.18 (-0.18, 0.55)	0.31
	P value †	< 0.001*	< 0.001*		
Cervical	T0	0.78 (0.07) A	0.73 (0.09) A	0.05 (-0.01, 0.11)	0.08
	T1	2.16 (0.19) B	2.04 (0.21) B	0.12 (-0.03, 0.27)	0.11
	T2	3.49 (0.30) C	3.26 (0.42) C	0.24 (-0.04, 0.51)	0.10
	T3	4.94 (0.48) D	4.69 (0.50) D	0.26 (-0.11, 0.62)	0.16
	Difference (T3-T0)	4.16 (0.45)	3.96 (0.54)	0.20 (-0.17, 0.57)	0.27
	P value †	< 0.001*	< 0.001*		
Difference	T0	0.16 (0.10) A	0.21 (0.05) A	-0.04 (-0.10, 0.02)	0.15
	T1	0.31 (0.07) B	0.25 (0.13) B	0.06 (-0.02, 0.14)	0.14
	T2	0.45 (0.09) C	0.52 (0.29) C	-0.07 (-0.23, 0.09)	0.38
	T3	0.60 (0.05) D	0.66 (0.31) D	-0.06 (-0.24, 0.12)	0.47
	Difference (T3-T0)	0.43 (0.08)	0.45 (0.33)	-0.02 (-0.21, 0.17)	0.83
	P value †	< 0.001*	< 0.001*		

*SD* Standard Deviation, *CI* Confidence Interval

#: Independent samples t-test was used

†: Repeated measures ANOVA was used

*Statistically significant at p value <0.05

A-D: Different letters denote statistically significant differences between timepoints within each group

### OIIRR

Malmgren Index scores for OIIRR of the maxillary canines in both groups SAG and MAG, on the experimental and control sides are represented in Table 5. Within each of the tested sides, a significant score change has been documented denoting an increased incidence and/or severity of OIIRR post-retraction on the experimental sides in the SAG and MAG groups (*p* < 0.001), and on the control sides in SAG (*p* = 0.08) and in MAG (*P* = 0.03).

**Table 5:** Comparison of root resorption scores between the experimental and the control sides in groups SAG and MAG

			Experimental side	Control side	*P* value
			N (%)		
SAG	Pre-	Score 0	10 (66.7%)	10 (66.7%)	1.00
		Score 1	5 (33.3%)	5 (33.3%)	
		Score 2	0 (0%)	0 (0%)	
		Score 3	0 (0%)	0 (0%)	
	Post-	Score 0	0 (0%)	5 (33.3%)	0.001*
		Score 1	3 (20%)	8 (53.3%)	
		Score 2	10 (66.7%)	2 (13.3%)	
		Score 3	2 (13.3%)	0 (0%)	
	P value		< 0.001*	0.008*	
MAG	Pre-	Score 0	11 (73.3%)	11 (73.3%)	1.00
		Score 1	4 (26.7%)	4 (26.7%)	
		Score 2	0 (0%)	0 (0%)	
		Score 3	0 (0%)	0 (0%)	
	Post-	Score 0	0 (0%)	6 (40%)	0.001*
		Score 1	3 (20%)	9 (60%)	
		Score 2	9 (60%)	0 (0%)	
		Score 3	3 (20%)	0 (0%)	
	P value		< 0.001*	0.03*	

Wilcoxon signed ranks test was used

*Statistically significant at p value < 0.05

**Fig. 9:**
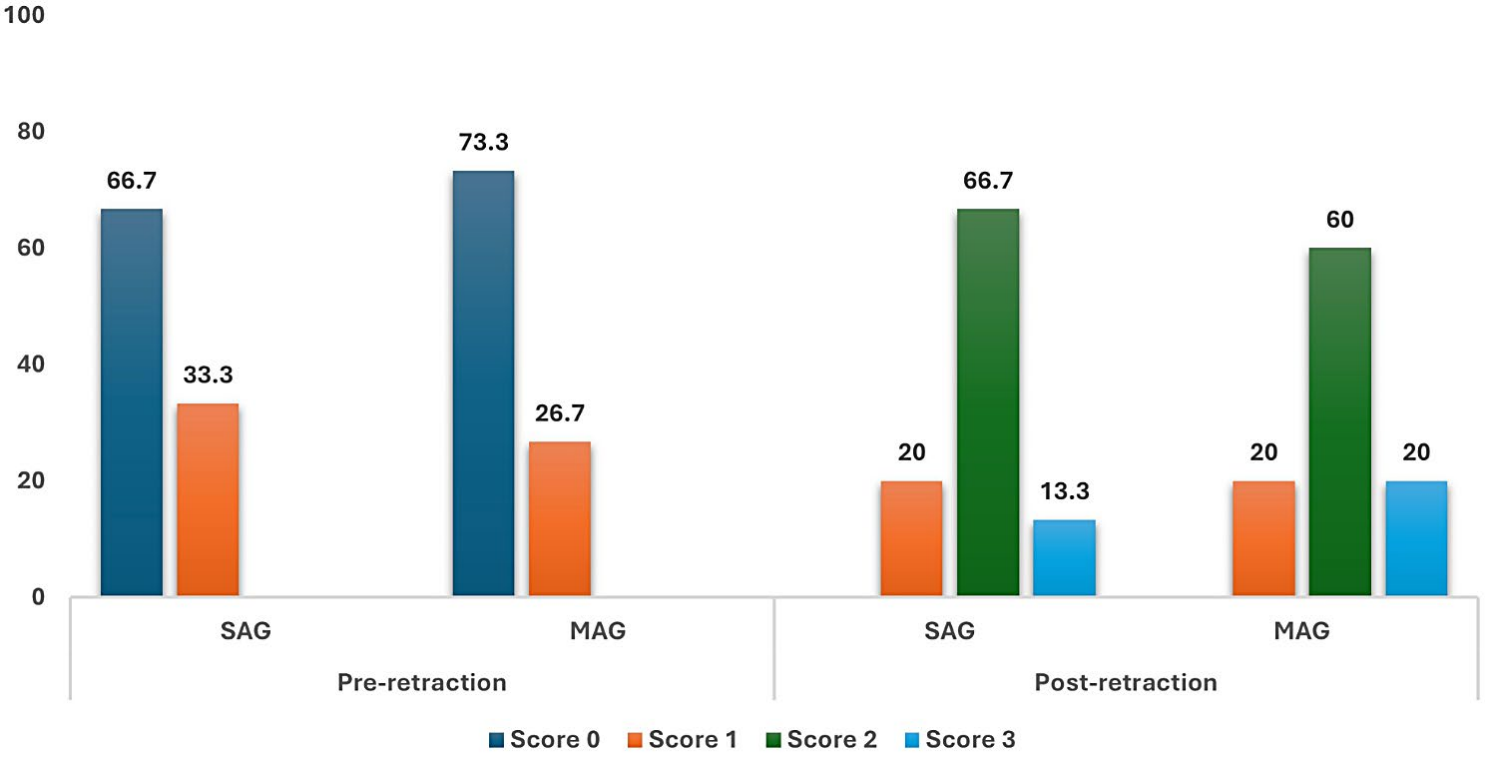
Comparison of root resorption scores on the experimental sides in groups SAG and MAG

Upon comparing the root resorption changes between the experimental sides in the two groups as displayed in Fig. 9, non-significant differences have been observed between them following canine distalization (*p* = 0.81).

## Discussion

With surgical methods being reported as effective acceleratory adjuncts to OTM, minimally invasive options are always advocated by both clinicians and patients. Hence, the objective of this study was to evaluate and compare the influence of single versus multiple piezocisions on the canine retraction rate. Moreover, canine tipping and root resorption were evaluated with both piezocision protocols. According to the reported results, the null hypothesis has been accepted as non-significant differences have been documented between single and multiple piezocision applications in all the measured outcomes, whether the amount of tooth movement, tipping, or the associated OIIRR.

The employed study design in the present investigation was a compound design randomized controlled trial (RCT), pertaining to RCTs being beheld as the benchmark for evaluation of intervention efficiency [32]. Furthermore, the split-mouth technique limited the influence of inter-subject variability, with the enrolled participants acting as their own controls, thereby decreasing the required sample size [33].

Extractions were scheduled at the beginning of orthodontic treatment, just after fixed appliance bonding, thus considerable time has been allowed between the extraction date and the onset of canine retraction. This sequence has been planned because extraction is considered a traumatic surgical procedure, that can induce RAP and alter the tooth movement rate, thereby obscuring the effect of the tested surgical intervention [34]. A similar precaution has been taken by several investigators [9, 35].

NiTi closed-coil springs were used to retract the maxillary canines in both groups, for the purpose of generating continuous forces throughout the 12-week assessment period [36]. Moreover, the medial ends of the third rugae were used a stable reference points for the measurement of canine retraction [37], as performed in former studies [6, 15].

The CBCT scans performed by the enlisted participants pre- and post-retraction (12-week interval) were imperative for assessing the influence of piezocision on root resorption. It is noteworthy to mention that high intra-observer and inter-observer reliability have been advocated regarding CBCT measurements in several investigations [38, 39]. Moreover, higher diagnostic accuracy has been reported with CBCTs when compared to periapical and panoramic radiographs with regards to the identification and diagnosis of root resorption [40, 41]. On another note, Malmgren index [29] has been used in the present study as a reliable scoring system for root resorption evaluation, as well in other studies [30, 42].

Results of the present study reported a significant increase in the amount of tooth movement on the piezocision sides in both the single and multiple application groups in comparison to the control sides, at all the assessed time points by approximately 62.5%. This resultant acceleration is mainly attributed to the RAP that has been induced by the surgical injury to cortical bone, and the consequent reduction in the bone resistance to tooth movement [16, 43]. Furthermore, in response to the employed selective decortication, an increase in the inflammatory markers together with an elevation in the cytokines’ levels take place, prompting the activity of osteoclasts and enhancing the bone remodeling process, finally resulting in acceleration of OTM [44, 45]. Findings reported in the present study are in agreement with those by Aksakalli et al [14], as well as Abbas et al [46], where piezocision was reported to significantly accelerate canine distalization into the extraction space by 1.5-2 times during the first three months of fixed appliance therapy.

Moreover, it has been observed that the greatest distances moved by the maxillary canines on the experimental sides in both groups were recorded in the 1st two months of treatment, followed by a significant decrease by the 3rd month, in contrast to the relatively constant rate of tooth movement on the control sides. However, despite the drop reported at T3, the distance travelled on the piezocision side was still significantly higher than that on the control side. A possible explanation has been provided by Wilcko et al [47, 48], where they stated that RAP is a unique phenomenon that exhibits a distinct pattern in its emergence and extent, with its onset taking place only a few days post-injury, reaching its peak after 4 to 8 weeks, and lasting for 2 to 4 months. A relatively similar pattern of tooth movement has been reported by Alfawal et al [15] with both piezocision and laser-assisted flapless corticotomies during canine distalization, and again by Jaber et al [49] with laser-assisted flapless corticotomy.

The relatively similar distances moved by the maxillary canines on the experimental sides in both groups with single and multiple applications do not support the theory recommending repeating the surgical injury in an attempt to re-induce the RAP, and consequently maintain the acceleratory impact on the teeth during orthodontic treatment. This theory has been tested by Sanjideh et al [50], where a second corticotomy procedure was performed after 4 weeks after treatment onset, and was found effective in accelerating OTM over a longer duration. With piezocisions, the same hypothesis has been investigated by Charavet et al [20], where one-stage versus two-stage piezocisions were compared, and repeated injuries were found to effectively re-activate RAP. However, when the same hypothesis was tested clinically in the present study, non-significant differences have been found between single and multiple piezocisions, thereby refuting the theory due to its clinical and statistical non-significance. This level of insignificance between both techniques could be explained by Wilcko et al [47, 48] as stated earlier, where they reported that RAP could last for up to 4 months, thus re-induction within 4 weeks is not needed.

Significant tipping of the maxillary canines in both groups has been reported on the experimental as well as on the control sides. This finding may be related to the direction of the force vector in the present study, which was in a distal and a relatively apical direction, since the NiTi coil springs were attached from the mini-screw head (8 mm apical to the apex of the interdental papilla, between the maxillary 2nd premolar and 1st molar), to the hook on the distal wing of the maxillary canine bracket. These findings are in accordance with those reported by Abbas et al [46] where significant tipping of the maxillary canines was noted after retraction, with both piezocision and corticotomy procedures, in comparison with the controls. However, the non-significant differences between both experimental sides (single and multiple piezocision) in the resultant tipping movement could be related to the non-significant differences between them in the rate of tooth movement that has been reported earlier as well.

Analysis of the root resorption scores in the present study revealed a statistically significant difference between the experimental and control sides in both groups SAG and MAG, with more resorption related to both the single and the multiple surgical interventions. Comparative findings were reported by Elkalza et al [24] and Patterson et al [27], where significant root resorption has been recorded with piezocision-assisted orthodontics. Conversely, others reported significantly less root resorption with piezocision in comparison to conventional orthodontic treatment [46].

Even though the induced RAP following surgical injury is known to increase alveolar bone turnover through stimulating the accompanying cellular activity could possibly reduce the incidence of root resorption due to the remarkable reduction in the pressure areas [51, 52], the precise association between alveolar bone density and OIIRR is quite perplexing. Contradictory findings have been reported in the literature regarding this issue, where some have suggested that the osteoporotic environment induced by corticotomy-related procedures favor bone remodeling around the roots [53], whereas others documented an increase in OIIRR with the increased bone turnover rate [54]. On a cellular level, three weeks post-corticotomy, an increase in osteoclast number has been noted in conjunction with a surge in the bone turnover rate, which was attributed to the RAP response [55]. On a biological level, Teng and Liou [56] found that bone remodeling markers from the gingival crevicular fluid, such as bone-specific alkaline phosphatase showed a constant increase throughout the experimental period following interdental cuts between the teeth in Beagle dogs. Furthermore, the experimental dogs did not encounter a systemic increase in bone turnover, as depicted through serum alkaline phosphatase levels, thus it has been concluded that the RAP response is experienced locally, and that the extent of the osteotomy is possibly directly related to the intensity of bone turnover and the associated osteoporotic changes. Accordingly, it could be argued that the increased clastic cellular activity during the enhanced turnover process, could possibly increase the expected amount of OIIRR.

Moreover, RAP has been reportedly associated with an increase in the local inflammatory response [51], with a consequent significant increase in inflammatory markers such as cytokines and chemokines at the injury site [9]. Since OIIRR is considered an inflammatory process, the elevated levels of inflammatory mediators induced by RAP and surgical injuries could be possible risk factors for root resorption [57].

### Study limitations

Limitations of the present study include the lack of assessment over a longer period and repeating the intervention after 2 or 3 months instead of every month, covering the entire orthodontic treatment duration. Therefore, future studies are recommended to extend past the canine retraction stage for a more comprehensive appraisal. Additionally, a larger sample size would have aided in the generalizability of the obtained results. It is also noteworthy to mention that the lack of operator blinding during the intervention could result in potential bias. Nonetheless, specific measures were taken to manage this downside, including the randomized subject allocation, as well as blinding of the operators during both the measurement and the data analysis phases.

Assessment of patient reported outcome measures are also advocated, such as pain, discomfort, functional limitation, periodontal side effects, in addition to patient acceptability to the repeated interventions. Moreover, measurement of maxillary canine tipping has been performed relative to the lateral incisor, hence, its assessment relative to a more stable reference point is recommended for a more accurate evaluation.

## Conclusions

1. Considering the present study’s 12-week interval, single and multiple piezocisions effectively accelerate OTM in comparison to conventional orthodontic treatment, with relative outcomes reported by both intervention frequencies. Accordingly, single piezocision is recommended as an adjunct to OTM.
2. Given the employed mechanics for canine retraction in the present study, significant tooth tipping accompanies accelerated OTM with both single and multiple surgical interventions, with comparable amounts using both protocols. Therefore, bodily tooth movement is less encountered in conjunction with piezocision-assisted orthodontics.
3. Incidence of OIIRR is significantly higher with both single and multiple piezocison applications in contrast to OTM solely, which could be related to the enhanced clastic cellular activity at the injury sites. Approximate OIIRR risks have been documented using both protocols.

## Data Availability

The datasets used and/or analyzed during the current study are available from the corresponding author on reasonable request.

## Abbreviations

OTM: Orthodontic tooth movement
RAP: Regional acceleratory phenomenon
OIIRR: Orthodontically induced inflammatory root resorption
NiTi: Nickel-titanium
3D: Three-dimensional
CBCT: Cone-beam computed tomography
FOV: Field of view
ICC: Intraclass correlation coefficient
SD: Standard deviation
RCT: Randomized controlled trial
